# Hydration and carbonation curing of high ferrite clinker (FePC) synthesized using EAF slag

**DOI:** 10.1007/s44242-024-00051-9

**Published:** 2024-12-04

**Authors:** Elijah Adesanya, Visa Isteri, Aniruddha Baral, Christiane Rößler, Theodore Hanein, Juho Yliniemi

**Affiliations:** 1 Faculty of Technology, Fiber and Particle Engineering Research Unit, University of Oulu, PO Box 4300, 90014 Oulu, Finland; 2 Faculty of Technology, University of Oulu, Process Metallurgy, 90014 Oulo, Finland; 3 School of Civil Engineering, University of Leeds, Leeds, LS2 9JT UK; 4 F.A. Finger-Institute for Building Material Science, Bauhaus-Universität Weimar, Weimar, Thüringen Germany

**Keywords:** Brownmillerite, Steel slag, Carbonation, Low-carbon cement, EAF slag, 钙铁石, 钢渣, 碳化, 低碳水泥, 电炉渣

## Abstract

This study explores the use of Electric Arc Furnace (EAF) slag as a sustainable alternative raw material in cement clinker production. The research demonstrates the synthesis of ferrite-rich clinker using EAF slag, achieving a clinker composition of 47% alite, 32% ferrite, and 20% belite while replacing 20% of clinker raw materials i.e. limestone, iron and silica source. The hydration behavior and influence of carbonation curing on the reactivity of the ferrite phase were assessed. Results show that the addition of 5% gypsum to the clinker enhanced the hydration rate of alite and ferrite phases, promoting the formation of portlandite, C-S-H and ettringite as the major hydration phases. Typical of ferrite-rich cement, Fe/Al-rich siliceous hydrogarnet was also identified as secondary hydration products of the ferrite phase, formed as a result of the reaction of katoite (formed from ferrite dissolution) with dissolved silica. However, prolonged carbonation exposure led to a decrease in the formation of the hydrogarnet and the decomposition of ettringite, but the mortar’s strength increased with increasing calcium carbonate formation.

**Supplementary Information:**

The online version contains supplementary material available at 10.1007/s44242-024-00051-9.

## Highlights

• EAF slag was used to produce hydraulic alite (47%)-ferrite (32%)-belite (20%) cement.

• The effect of carbonation and gypsum on hydration was studied.

• Hydration of ferrite led to formation of Fe/Al-rich siliceous hydrogarnet.

• Leaching of Cr, Mo, and V originating from slag was under limits after hydration.

**Supplementary Information:**

The online version contains supplementary material available at 10.1007/s44242-024-00051-9.

## Abstract (zh)

本研究探讨了电炉渣作为可替代原材料用于制备硅酸盐水泥熟料的可行性。结果表明，使用电炉渣替代20%的传统原料（如石灰石、含铁和硅原料）合成了高铁相熟料，其中硅酸三钙47%、铁相32%、硅酸二钙20%。本研究还分析了铁相的水化与碳化养护对其反应活性的影响。结果表明，在熟料中添加5%的石膏提高了硅酸三钙和铁相的水化速率，促进了氢氧化钙、C-S-H和钙矾石等主要水化产物的生成。作为富铁相水泥的典型特征，富铁/铝硅质水榴石为铁相的二次水化产物，由水钙铝榴石（铁相溶解形成）与溶解的二氧化硅反应形成。然而，延长碳化时间会减少水榴石的生成和钙矾石的分解，但砂浆强度能够随着碳酸钙含量的提高而增强。

## Introduction

The United Nations environment programme (UNEP) on emissions gap stated that, to limit global warming to 1.5 °C, current global greenhouse gasses must be cut by 45% by 2030 [1]. To achieve this objective, all anthropogenic greenhouse gas (GHG) emitting industry would need to reduce their environmental footprint. In 2013, The European Cement Association (CEMBUREAU) identified five potential parallel routes towards reducing cement carbon footprint by 32% by 2050 compared with 1990 levels. Two of these routes identified are towards carbon reduction via resource efficiency (i.e., raw material substitution), and carbon sequestration and reuse. The use of alternative raw materials in cement production also offers the advantages such as the reduction of quarrying for materials (limestone, clays, and chalk etc.), lower carbon emissions if the alternative materials are uncarbonated, and landfill diversion if waste materials are used.

**Fig. 1:**
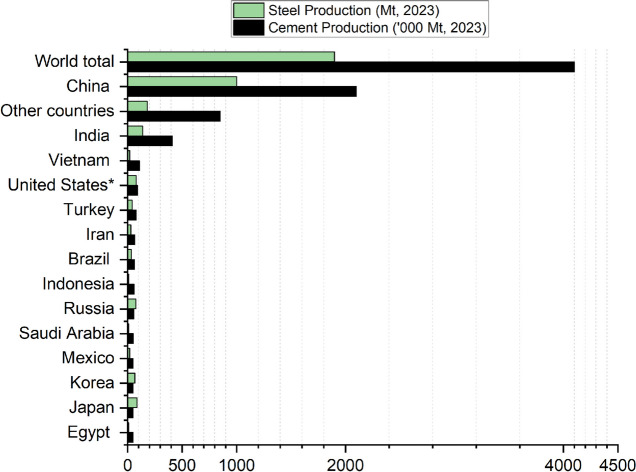
Global cement and steel production in 2023(* including Puerto Rico). Global steel slags production was estimated based on this as it constitutes 10%–15% of steel production

Slags generated during steelmaking process are considered as a good alternative raw material with similar components as traditional raw feedstock in clinker production as they contain similar element oxides such as CaO (22%–60%), SiO_2_ (6%–34%), and Fe_2_O_3_ (10%–40%) [2]. In the European Union alone, about 17 million tons of steel slags were produced in 2021, and global production is estimated at 280 million tons annually [3, 4]. These slags which includes electric-arc furnace slags (EAF), basic-oxide furnace slags are underutilized due to their poor hydraulic properties and their use as supplementary cementitious materials are however limited. One of the most common limitations of using waste materials or residues in cement is their availability. Steel slags are widely available regionally across the world as residues from steel production (see Fig. 1). China as one of the biggest consumers of cement and concrete produces in excess of 100 million tonnes of steel slags annually [5]. Furthermore, it is evident that countries that produce a lot of cement also produce a lot of steel (Fig. 1). In Northern Europe there is an urgent need in finding pathways to utilize steel slags due to steelmaking process transitioning to direct hydrogen reduction (DRI) of iron ore (shutting down blast furnaces and replacement with DRI already in 2025–2035). The production of EAF slag is projected to increase and be available in large volume in the future. Hence, for cement production, the replacement of limestone with these iron and calcium rich slags has great potential to reduce CO_2_ emissions related to the clinker feedstock and increase steel slags valorization.

It is a promising pathway to increase the ferrite phases in the clinker, among which the most common are brownmillerite (C_4_AF) and srebrodolskite (C_2_F) [3], endmembers of solid solution series. The formation of these phases has been reported to also improve the burnability of the clinker by reducing clinkering temperature from 1450 ºC to 1350 ºC [6, 7]. Elakneswaran et al. [6] reported a 5% reduction in energy consumption when ferrite-rich Portland cement was produced at 1350 ºC. This saving is sizable considering that a modern cement plant consumes about 110–120 kWh per ton of cement [8, 9]; the lower temperature required is also favorable towards reducing energy consumption during clinkering. An additional incentive to increase the ferrite phase (high Fe/Al) in cement is to limit the formation of tricalcium aluminate content towards improving the sulfate resistance of cement. Shao et al. [10] studied the effect of high Fe/Al of ferrite phase in cement on its sulfate resistivity, results from the study showed enhanced sulfate resistance when compared to ordinary Portland cement (OPC). This is consistent with results also reported by Yang et al. [11]. High-ferrite cements are similar to cements used in oil-well construction, and research suggests that increasing the ferrite content in oil-well cement enhances the strength, durability, and toughness of the hydrated cement [12, 13].

Iron-rich EAF slag as one of the raw feedstocks for different types of cement clinker production has been reported such as in sulfoaluminate belite cement [14, 15], calcium ferroaluminate belite cement [16], and in Portland cement (PC) [17]. The utilization of steel slags as alternate raw materials for clinker production has the potential to reduce limestone, iron ore, and energy consumption. In a study by Gao et al. [18], between 6.9% – 7.4% of steel slag was used as alternative calcium and iron source in cement production, they indicated that iron-ore used in clinkering can all be replaced and some portion of limestone using steel slags. By using steel slag in cement production, Gao et al. [18] reported a decrease in the total power emissions by 1.51 kg/t. In a recent study on material substitution in cement production, EAF slag in molten state in the electric arc furnace was synthesized with recovered cement paste to produce cement like materials. The formation of alite, belite, tricalcium aluminate and gehlenite were reported and no ferrite phase was formed [19].

Cements with high ferrite content commonly show a lower early-age strength gain, and additives such as tertiary alkanolamines [7] and soda ash [20] have shown to increase the ferrite reactivity. In addition, potential ways to enhance the ferrite reactivity is to carbonate it, using calcium ions dissolving from ferrite with CO_2_ to form calcium carbonate. This process reduces the calcium ion concentration in the solution and on the surface of the ferrite, in turn this may lead to an increase of the dissolution rate and reactivity of ferrite to form Fe-gel, siliceous hydrogarnets or Fe-hydroxides. Specifically, for C_3_S-C_4_AF systems, the effect of carbonation on hydration products and properties for fresh state mortars has not been reported thus far. Together with sustainable cement production, the cement and concrete carbon capture technique is also recognized as a viable route towards low or net zero carbon cement production [21]. This technique has gained traction and have been successfully scaled up especially in precast concrete and masonry [22]. While steel slag is often recycled for various uses, it can contain several contaminants and heavy metals such as chromium, lead, and arsenic that may pose environmental and health risks [23]. The stability and activity of these heavy metals in the cement clinker are relevant study pathways.

In this current study, the objective is to utilize 20 wt.% of EAF slag as alternative raw feedstock in cement clinker production with the goal of synthesizing C_3_S-C_4_AF-C_2_S rich clinker system. The experimental studies initially examined the hydration of clinker both with and without gypsum, and then the effect of carbonation on the hydrated products towards enhancing the reactivity of the C_4_AF phase. X-ray diffraction (XRD), isothermal calorimetry, field emission scanning electron microscopy (FESEM) and thermogravimetry analysis (TG/DTG), were used to analyze the hydration of the C_3_S-C_4_AF-C_2_S cement, the effect of carbonation on the C_4_AF phase, the hydrated paste phase assemblage, and microstructure. Compressive strength developments, density, mercury intrusion porosimetry (MIP), and leaching tests were determined to analyze the evolution changes and stability in the carbonated cement samples in comparison with the reference non-carbonated cement.

## Materials and experimental methods

### The alite–ferrite phase clinker formulation and synthesis

A ferrite-rich clinker with a target composition of 25 wt.% ferrite (C_4_AF) phase, was synthesized using a mix of EAF slag (20 wt.%) as alternative feedstock, calcium carbonate, kaolin-rich clay, and silicon oxide. The fraction of EAF slag used was based on the calculation of the targeted mineralogy composition (25% of ferrite phase and 50%–75% of alite). The targeted clinker mineralogy was calculated using stoichiometric chemical compositions of the target phases. The EAF slag used in this study was demagnetized and collected from Magsort Oy (Finland), calcium carbonate (CaCO_3_) was supplied by VWR (Finland), silicon (IV) oxide (SiO_2_) was supplied by ThermoFisher (Germany), and the Kaolin clay was supplied by Sigma-Aldrich (Germany). The slag was demagnetized to recover valuable metals in its composition. The chemical composition of EAF slag and the kaolin clay (Table 1) were characterized using X-ray fluorescence (XRF) spectroscopy (PANalytical Omnian Axiosmax). The combined value of CaO, Fe_2_O_3_, and SiO_2_ (~ 81%) in the slag meets the requirement value for industrial waste chemical composition in cement kiln, e.g., in China [24]. Gypsum used for the cement hydration was supplied by VWR (CaSO_4_·2H_2_O; powder, 99%; CAS: 10,101–41-4).

**Table 1:** Chemical composition of the starting materials (%)

Oxides	CaO	Al_2_O_3_	SiO_2_	MgO	Fe_2_O_3_	P_2_O_5_	SO_3_	Cr_2_O_3_	TiO_2_	Others
EAF slag	39.1	7.6	14.8	8.0	26.6	1.2	0.6	0.07	0.6	1.4
Clay	0.1	36.8	53.2	0.3	1.2	0.2	-	-	0.1	8.1

### Clinker preparation

The raw mixes of EAF slag (20%), calcium carbonate (67%), kaolin clay (5%) and silicon dioxide (7%) were homogenized in a ball mill for 30 min and then granulated in a granulation disc (dia. of 40 cm and depth of 8 cm, rotated at 35 rpm) with a tilting angle of 45°. The granules were then dried at 100 °C for 48 h, before clinkering. The clinker was produced from the granules in 1 kg batches in alumina crucibles. The crucibles were placed to preheated muffle furnace (800˚C); the temperature is temporary descending when crucible is placed into the furnace and was allowed to stabilize back to 800 ˚C and kept at 800˚C for 30 min. After preheating the temperature was elevated to 1400˚C at a rate of 4 ˚C/min (~ 2 h 30 min). The firing stage at 1400 ˚C was 1 h and after that, the samples were directly taken out from the furnace and cooled at room temperature on a steel slab. The cooling was improved by using compressed air gun until the clinker was no longer glowing. The clinker granules were crushed using a laboratory jaw crusher (Retsch BB 51) before they were milled using a Germatec ball mill containing 30 (50 mm), 100 (33 mm), and 60 (20 mm) balls until median particle size (d_50_µm) below 10 µm was reached. The particle size distribution was analysed using a Beckmann Coulter LS 13320 laser diffraction particle size analyzer in isopropanol media.

### Paste and mortar’s preparation

Paste samples used for characterization were prepared according to Table 2 without sand, using a Helkama (Finland) hand mixer. Mortar samples were prepared using a Controls Automix (Italy) conforming with EN 196–1 standard procedures, using the mix composition in Table 2. After mixing and casting, all samples were precured for 20 h at 24 °C and 60% relative humidity (RH) to remove excess moisture before they were transferred to a carbonation chamber (Table 3). A reference sample without carbonation (FePC_0h) was after pre-conditioning immersed in water for further curing ages 7 and 28 days. The samples were exposed to 20% concentration of CO_2_ at 60 ± 10% RH and 25 °C in the chamber at varying reaction times 6 h, 24 h, and 72 h and tagged FePC_6h, FePC_24h and FePC_72h respectively. Thereafter each sample was removed after the reaction time and immersed in water for further curing until 7 and 28 days after mixing (including carbonation curing). At 7 and 28 days, the hydration of the paste samples used for characterization were stopped through solvent exchange using isopropanol. Initially, the samples were immersed in isopropanol for 30 min and the solvent discarded. The samples were then immersed in fresh isopropanol for 24 h and then removed and dried at 40 °C for 4 h. This same procedure of solvent exchange was repeated two more times after cooling. Then the samples were stored in a desiccator.

**Table 2:** Mix composition for paste and mortar samples (g)

Sample	AF clinker	Gypsum	Water	Sand
Paste	427.5	22.5	180	-
Mortar	427.5	22.5	180	1 350

**Table 3:** Pre-conditioning, carbonation and curing procedures

Sample name	Pre-conditioning	Carbonation conditions	Carbonation duration (h)	Further curing
FePC_0h	24 °C, 60% RH, 20 h	-	0	7, 28 days (incl. carbonation)
FePC_6h		60 ± 10% RH, 25 °C, and 20% CO_2_	6	
FePC_24h			24	
FePC_72h			72	

### Test methods

#### Isothermal calorimetry

The heat released during the hydration of the cement pastes for sample with and without gypsum were recorded using a TAM Air isothermal microcalorimeter at 20 °C. The paste sample was mixed in-situ for 2 min using a TAM Air admix ampoule at 60 rpm. The generated heat flow was normalized to the binder mass in the paste.

#### X-ray diffraction

The minerology of the milled clinker and the hydrated cement pastes finely ground in an agate mortar were determined through XRD, performed using Rigaku SmartLab 9 kW X-ray diffraction equipment with Co Kα radiation at a scan rate of 4°/min in the range 5°–70° (2θ), and 0.02°/step. Phase identification was done using “X’pert HighScore Plus” (PANalytical software). In-situ hydration of the clinker was monitored using a PANalytical X’pert^3^ that operates using a Cu source (Kα1 = 1.540 598 0 Å) at 45 kV and 40 mA. The diffraction pattern was collected from 2θ values ranging from 5° to 55° with a step size of 0.013° and a total scan time of 16 min. Rietveld refinement of the phases was done using X'pert HighScore Plus analytical software, then the degree of hydration (DOH) was estimated based on Eq. 1.

1 $$DoH=\frac{{M}_{i}-{M}_{t}}{{M}_{i}}$$

where *M*_*i*_ is the weight fraction of the anhydrous phase, *M*_*t*_ is the weight fraction at a time after hydration, normalized to 100 g of solid content in the paste sample.

#### Scanning *electron* microscopy (SEM)

The microstructural evolution of the hydrated cement pastes with and without carbonation were investigated at 7 and 28 days of hydration using a Zeiss Ultra Plus SEM with an accelerating voltage of 15 kV and working distance 8.5–9 mm, equipped with energy dispersive X-ray (EDX) spectroscopy. Before scanning, the cut samples were impregnated under vacuum with epoxy resin, and the cross section polished using diamond pastes and ethanol. Carbon coating was sputtered on the polished sample surface for conductivity.

For the SEM phase mapping, samples of 7-day and 28-day hydrated and carbonated cement pastes were embedded in epoxy resin. Sample polishing was carried out using an automatic polishing device (TF250, JeanWirtz, Germany) and diamond oil pastes (MetaDi II, Buehler, US). Successive polishing with oil-based diamond pastes of sizes 15, 9, 3, 1 and 0.25 µm was applied. To achieve electric conductivity, the polished sections were coated with approximately 8–10 nm carbon. SEM–EDX investigations in high vacuum were carried out by using a Helios G4 UX (ThermoFisherScientifc, Netherlands) equipped with a field emission gun. EDX spectrometer (X-Max80, Oxford Instruments, UK) is attached to the SEM. Quantification of EDX spectra was carried out using the Oxford Instruments standard-less quantification procedure (using remote standards) including matrix correction based on the Extended Pouchou and Pichoir (XPP) model as included in the software Aztec 5.0 (Oxford Instruments, UK). Phase maps were obtained using the cluster algorithm included in Aztec 5.0 and subsequent manual modification of results to limit number of phases (i.e. merging of phases with similar composition and excluding phases with low number of pixels).

#### Thermogravimetry analysis (TGA)

TGA/DTG analyses for the hydrated samples were analysed using SDT-650 by TA instrument with autosampler. The ground paste samples weighing between 9–12 mg in an alumina crucible were then heated from 38 °C to 1000 °C at a heating rate of 10 °C/min in a nitrogen atmosphere with a flow rate of 100 ml/min.

#### Leaching and hexavalent chromium [Cr(VI)] determination

Leachability analysis of the anhydrous FePC, hydrated and carbonated FePCs were determined based on SFS-EN 12457–2 [25]. The procedures follow a one stage batch test at a liquid to solid ratio 10 l/kg for materials with particle size below 4 mm (without or with size reduction). The samples were agitated for 24 h and then filtered. The pH of each sample was also recorded, and the concentration of the various elements was then determined using ICP-OES. The amount of water-soluble Cr(VI) in the clinker was determined via colorimetric method using filtered extracts of fresh FePC pastes in accordance to standard procedures in SFS EN 196–10 [26]. The concentration of Cr(VI) was then measured using VWR UV-6300PC double beam spectrophotometer at 540 nm. Calibration standards were in the range 0.1 to 1.5 mg/L.

#### Mercury intrusion porosimetry

Mercury intrusion porosimetry was used on the hydration stopped samples to measure the pore size distribution. MIP measurements were performed using a Micromeritics Autopore 9600 Mercury Porosimeter, with intrusion and extrusion pressure up to 60 000 psia. The contact angle of mercury was assumed to be 130°, and surface tension of mercury was taken as 0.48 N/m.

#### Compressive strength test

The compressive strengths of the composite mortars with dimension 40 mm × 40 mm × 160 mm were determined at 7 and 28 days using a Toni Technik strength testing machine (Germany) with a maximum load cell of 3 000 kN and a force speed of 2.4 kN/s according to specifications in EN 196–1 standard. Four replicates were mechanically crushed, and the average value was reported as compressive strength.

#### Selective dissolution

Selective dissolution of the hydration products (i.e. C-S-H, AFt, AFm, CH) in the pastes for the non-carbonated and carbonated samples was undertaken using the methods proposed by Dilnesa et al. [27]. This technique helps in enriching minor phases such as siliceous hydrogarnets that may not be identified prior to extraction. Hence, 5 g of each paste sample was added to a solution containing 20 g of Salicylic acid and 300 ml of methanol, the suspension was stirred for 2 h in a conical flask with the aid of a magnetic stirrer. The suspension was then allowed to settle for 15 min and vacuum filtered through 0.45 µm filter. The residue remaining on the filter was rinsed with methanol and dried at 90 °C for 45 min. To determine the siliceous hydrogarnet and other enriched phases after dissolution, the residues were analyzed via TGA.

## Results and Discussion

### Clinker characterization

**Fig. 2:**
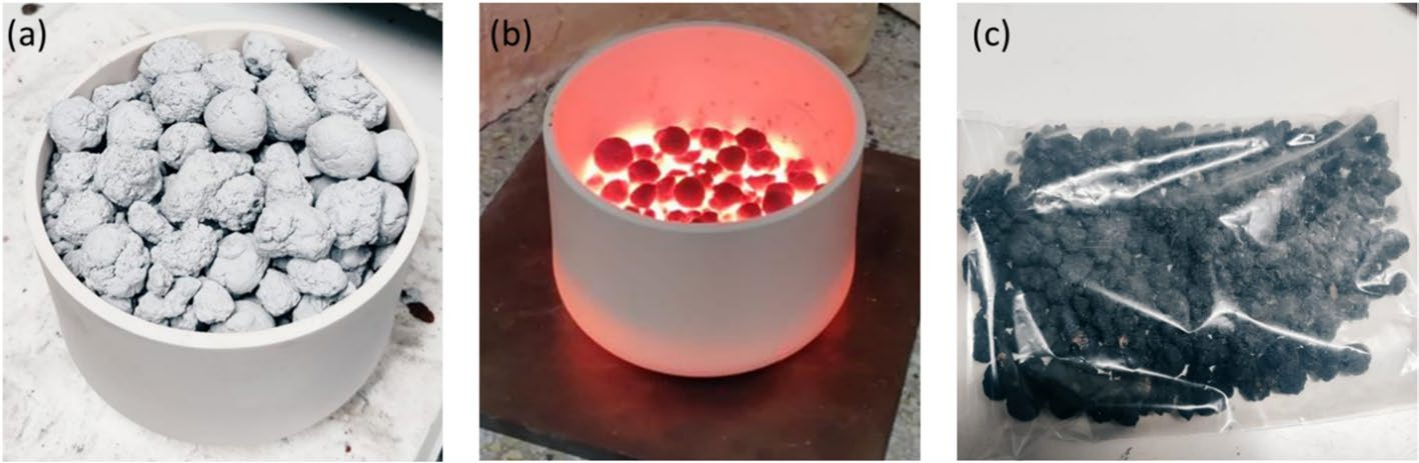
Depiction of the granulated raw materials (**a**) before clinkering, (**b**) cooling (before cooling with an air gun), and (**c**) unground, cooled clinker

The granulated raw materials used for the clinker before and after clinkering are shown in Fig. 2. The median particle size (d_50_) for the milled clinker was 6.28 µm, while approximately 90% of the clinker particle sizes were below 20 µm and the density was 3.3174 g/cm^3^.

**Fig. 3:**
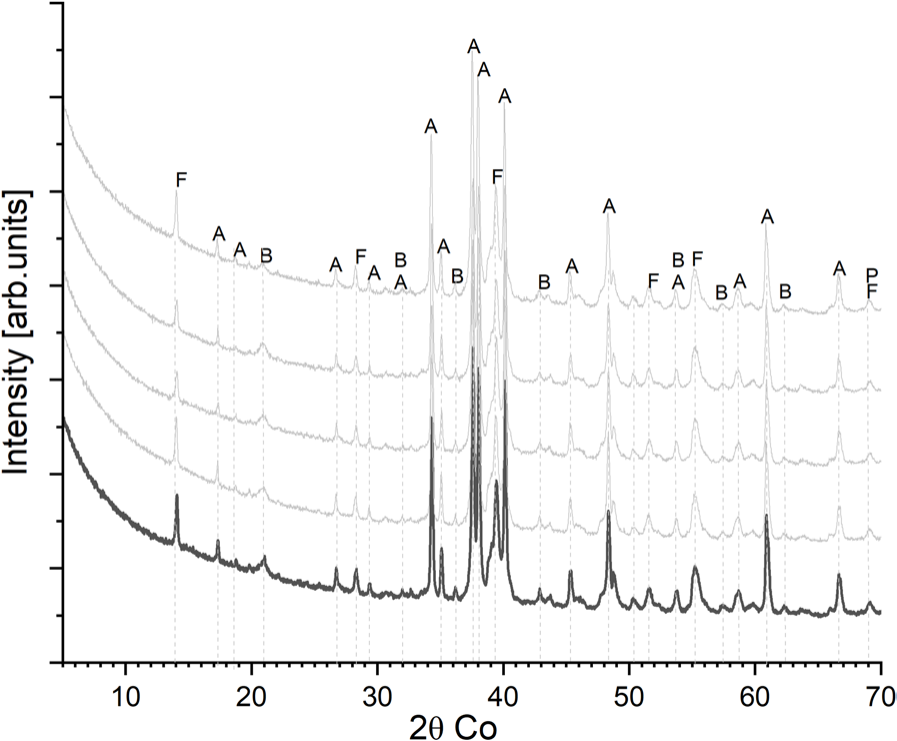
XRD diffractogram patterns of clinkers produced in four batches and the aggregated clinker (highlighted). The strongest peaks of major phases identified as follows A: Alite (C_3_S – Ca_3_SiO_5_); B: Belite (C_2_S – Ca_2_SiO_4_); F: Ferrite (C_4_AF–Ca_2_(Al,Fe)_2_O_5_); and P: Periclase (MgO)

The XRD pattern of the milled clinker is presented in Fig. 3, and the average quantified mineralogy of the clinkers using the Rietveld method is shown in Table 4. The actual content of the phases varied from the targeted content; this is caused by the variations from the stoichiometric calculations used for the targeted phases. To ascertain the consistency of the clinkering procedure, XRD patterns of four different batches of clinker produced on different days using the same parameters and conditions were analyzed and compared with the homogenized clinker (highlighted). The occurrence and intensities of the crystalline peaks were similar for all clinkers and the homogenized clinker (highlighted pattern). The main phases identified in the clinkers are alite (C_3_S, PDF# 04–014–9801) [28], belite (C_2_S, PDF# 04–013–6291) [29], characteristics peaks of ferrite (C_4_AF, PDF# 04–014–6637) [30], and traces of periclase (MgO, PDF# 01–085–5626). Tricalcium aluminate (C_3_A) crystalline patterns were not detected in this cement; hence, the cement can be classified as a sulfate-resistant Portland cement (CEM I SR-0) based on cement specifications [31]. The actual chemical composition of the clinker is listed in Table 5.

**Table 4:** Average Rietveld quantification of the mineralogical content of the clinkers: Alite (C_3_S – Ca_3_SiO_5_), Belite (C_2_S – Ca_2_SiO_4_), ferrite (C_4_AF–Ca_2_(Al,Fe)_2_O_5_), lime (CaO) and Periclase (MgO) (wt. %)

	C_3_S	C_4_AF	C_2_S	CaO	MgO
FePC	47.0 ± 1.9	31.8 ± 1.5	19.7 ± 3.0	0.2 ± 0.2	1.3 ± 0.4

**Table 5:** Calculated and actual chemical composition of the Fe-rich clinker (%)

Oxides	CaO	Al_2_O_3_	SiO_2_	MgO	Fe_2_O_3_	P_2_O_5_	SO_3_	Cr_2_O_3_	TiO_2_
Calculated	66.8	5.3	19.7	2.4	8.2	0.4	0.2	0.02	0.2
Actual	65.2	5.6	18.6	2.3	7.8	0.2	0.2	0.03	0.2

**Fig. 4:**
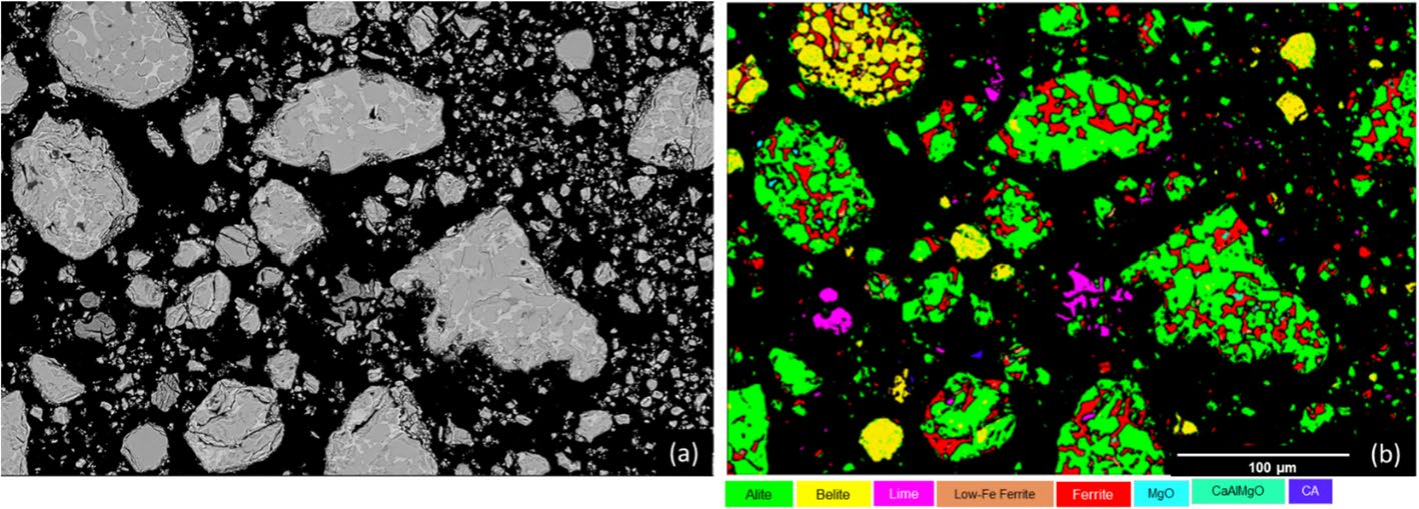
**a** The SEM-BSE image and (**b**) the corresponding phase map of the clinker, revealing the major phases: alite, belite, ferrite/lowFe ferrite, and minor phases of Ca-Al–Mg-O, lime, and CA

To verify XRD phase identification and to analyse chemical composition of clinker phases, SEM–EDX was performed. The BSE image and corresponding clinker phase map are shown in Fig. 4. Phase mapping confirmed that three major phases, i.e. tricalcium silicate (C_3_S-alite), tetracalcium aluminoferrite (C_4_AF-ferrite), and dicalcium silicate (C_2_S-belite), were formed in the clinker. The phase map revelas two ferrite compositions, which are of major interest in this study and which can be discriminated by their F/A ratio (Table 6). The low Fe ferrite is a minor constituent of the clinker. The high Fe-ferrite phase, can be defined using the formula Ca_2_(Fe_1-x_Al_x_)_2_O_5_ in the absence of other oxides, where 0.0 < x < 0.7 [32]. This ferrite (Fig. 4.) represents a chemical composition close to that of the stoichiometric brownmillerite phase C_4_AF (Al/Fe molar ratio = 1.3). The second observed ferrite (low ferrite), posses higher amount of Ca, Al and lower amount of Fe. The difference in the F/A ratio between these two ferrite phases may be caused by the different cooling speeds, which is an important factor in the F/A ratio of ferrite [33]. A higher alumina content in the ferrite phase (lower F/A ratio) generally increases the reactivity of the ferrite phase [34]. Calcium aluminate (CA) was spotted in minimal area of the phase map, and consistent with XRD results.

**Table 6:** Elemental composition (given in oxide wt.%) of the clinker phases from SEM–EDX spot analyses (Fig. 3)

	Na_2_O	MgO	Al_2_O_3_	SiO_2_	P_2_O_5_	SO_3_	CaO	TiO_2_	MnO	Fe_2_O_3_
Alite	0.0	1.4	1.3	24.3	0.4	0.1	71.2	0.2	-	1.2
Ferrite	0.0	3.2	18.4	4.7	0.1	0.2	49.6	0.6	0.8	22.3
Low Fe-Ferrite	0.1	1.5	24.5	8.3	0.1	0.1	58.8	0.2	-	6.3
Belite	0.1	0.5	1.7	30.7	0.5	0.7	63.3	0.3	-	1.9
MgO	-	97.3	0.3	0.5	-	-	1.8	-	-	-
Lime	-	1.5	0.2	0.4	0.2	-	97.7	-	-	-
Ca-Al–Mg-O	0.2	16.9	22.8	5.6	-	0.1	47.9	-	-	6.5
CA	-	4.7	44.5	5.2	-	-	36.4	-	-	9.1

### Kinetics of hydration: effect of gypsum on clinker hydration

**Fig. 5:**
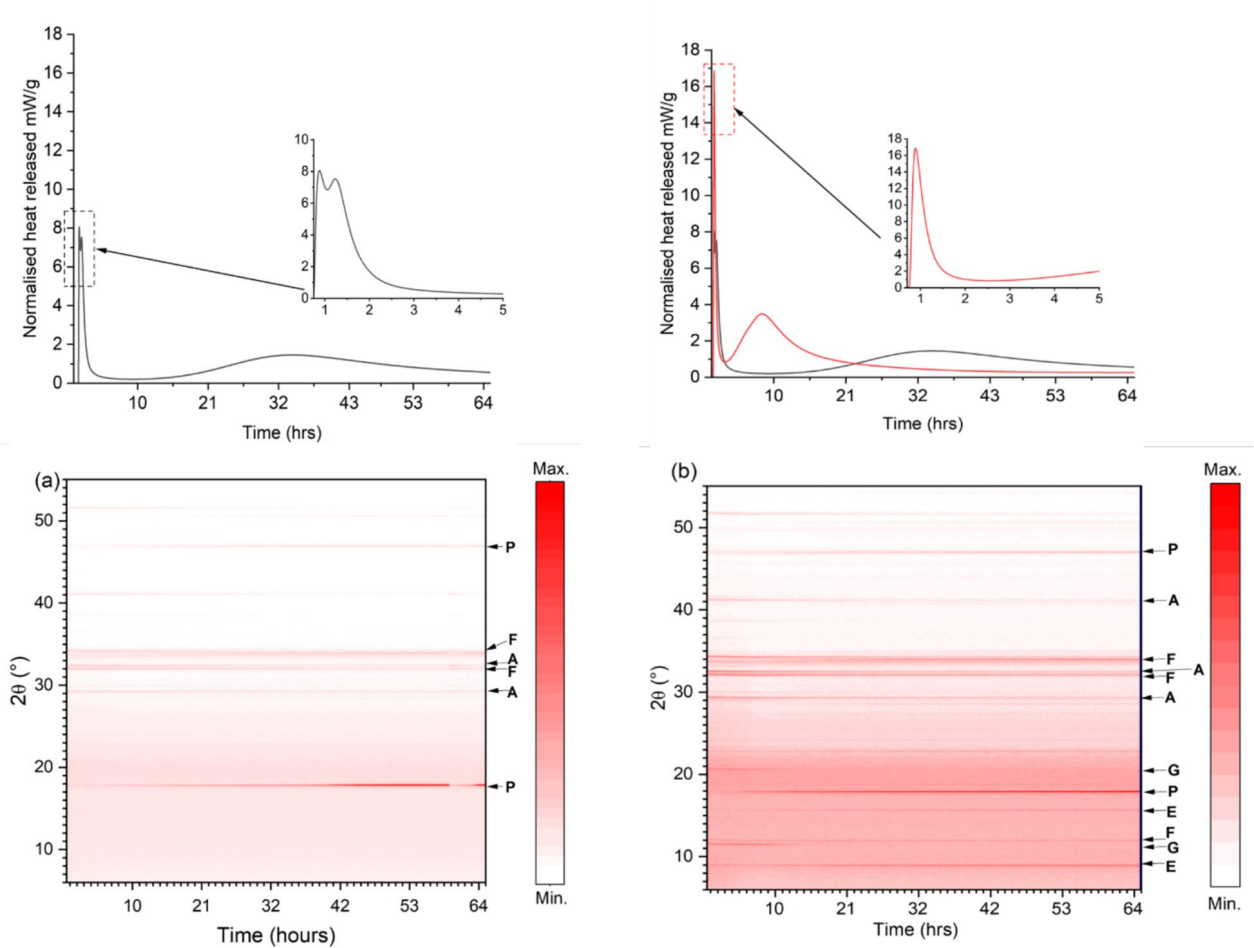
Heat evolved during hydration and in-situ XRD measurements over time of (**a**) FePC without gypsum and (**b**) FePC with gypsum. Where A: Alite, F: Ferrite, E: Ettringite, G: Gypsum, and P: Portlandite

The heat released during 65 h of hydration for the FePC clinker in the presence and absence of gypsum, is shown in Fig. 5 as obtained via isothermal calorimetry. In addition, for the same hydration period the clinker phase dissolution and formation of hydration products as revealed by in-situ XRD measurements are shown in Fig. 5 (below). Both samples exhibit typical calorimetric curves representative for cement systems. Five calorimetric stages can be distinguished from the two curves [35]. The initial heat released was assigned to wetting and dissolution (i.e., Al^3+^, Ca^2+^, SO_4_^2−^, and Fe^3+^) of the cement particles, during which ettringite (initial formation) and/or Fe hydroxides start to precipitate. During this period, ettringite was detected in the XRD pattern. Some studies have suggested the early formation of Fe hydroxides (ferrihydrite) during the first hours of hydration [27, 36]. In the presence of gypsum, the initial peak was approximately twice as high as the corresponding peak without gypsum. The induction period after the initial peak was shortened in the presence of gypsum, while a dormant period up to 15 h characterizes the hydration of the mix without gypsum. This can be ascribed to the increased formation of ettringite (possibly Fe-ettringite) and enhancement of alite hydration during this period. It is worth stating that the hydration of FePC with gypsum as observed visually sets under 12 h while FePC without gypsum sets between 30–48 h. The second peak is assigned to the heat released from further dissolution of C_3_S and formation of CH and C-S-H. Regarding the acceleration peak, this region coincides with the depletion of gypsum in the XRD patterns which suggest the second formation of ettringite or renewal of ferrite hydration. The intensity of the ettringite in the XRD patterns was maintained thereafter which may suggest minimal or no further formation of this phase. Although ferrite has lower reactivity, the change in its hydration during in-situ XRD is inconclusive due to low intensity.

### Impact of carbonation on phase assemblage and pore structures of FePC

#### XRD

**Fig. 6:**
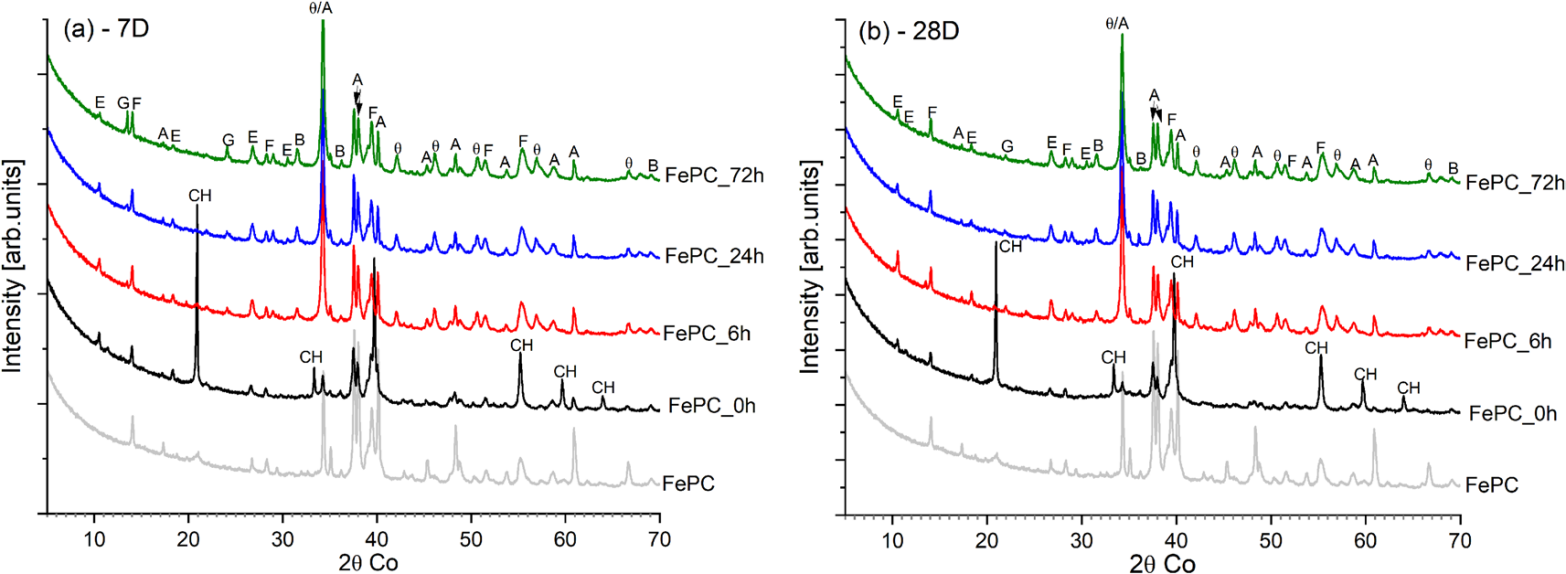
XRD patterns of the anhydrous FePC, hydrated and carbonated FePC at (**a**) 7 days and (**b**) 28 days. Where A: alite, B: belite, F: ferrite/brownmillerite, E: ettringite, G: gypsum, CH: portlandite, and Ꝋ: calcite

**Fig. 7:**
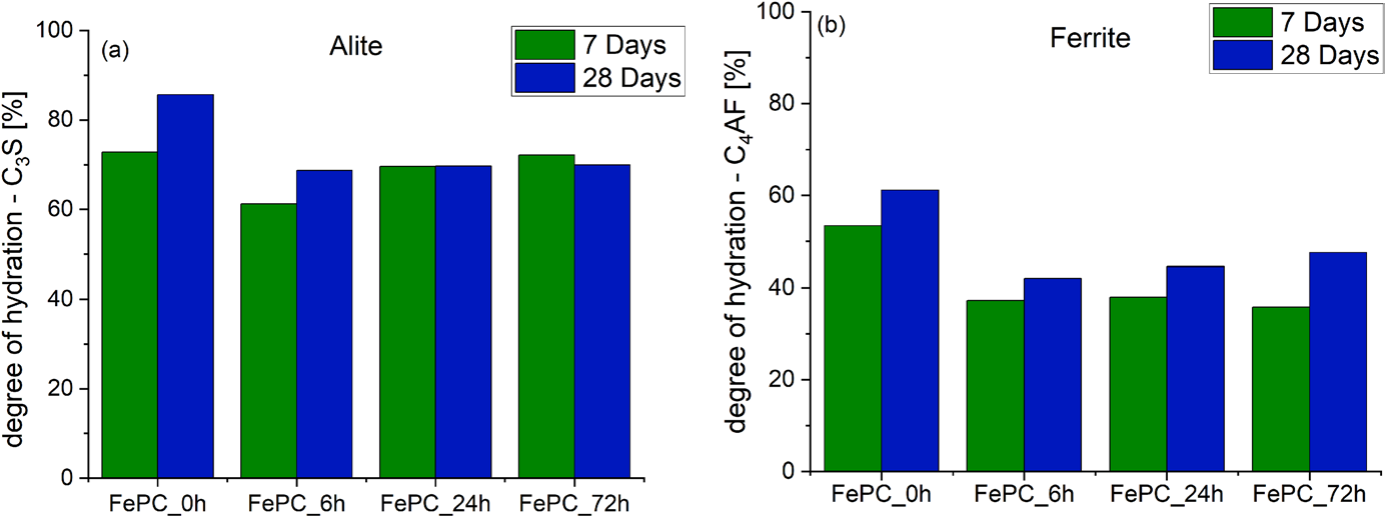
Degree of hydration (**a**) alite, (**b**) belite, and (**c**) ferrite phases determined by XRD Rietveld analysis. Accuracy of the measurements is assumed to be ± 2%

After 7-days and 28-days hydration, the XRD patterns for the FePC pastes that underwent carbonation for 0, 6, 24 and 72 h were recorded and are shown in Fig. 6. Results reveal the presence of CH, and ettringite as the major crystalline phases in the FePC_0h pastes (Fig. 6a), whereas CH is absent in all carbonated samples (Fig. 6b). Characteristic peaks of the semi-crystalline C-S-H phase were not detected hence its formation was characterized as part of the amorphous phase in the samples. The amorphous content characterized (See Fig.S2) is also attributed to include formation of silica gel, and amorphous calcite [37]. Unreacted cement minerals can still be found at both ages analysed. Their degree of hydration (DOH) based on the Rietveld quantification was further analysed as shown in Fig. 7. The DOH of alite was 15% higher in the FePC_0h sample at 28 days when compared to the carbonated samples. This is ensued from the continuous hydration of alite and formation of hydration products i.e. C-S–H in the FePC_0h sample. While during accelerated carbonation, the carbonated phases can form a layer on the unreacted alite phase, while CH carbonation produces CaCO_3_ acting as a barrier to further formation of C-S-H [38, 39]. However, comparing the carbonated samples, both FePC_24h and FePC_72h show a higher DOH of alite than FePC_6h at 7 days. Alite is known to be reactive to CO_2_ [28], which may imply that in addition to the hydrated alite, an increased carbonation of alite with increased exposure time may have occur. Gypsum was detected in the carbonated FePC, which is due to prolonged carbonation of ettringite [40, 41].

In contrast to alite, the DOH of ferrite (Fig. 7b) was considerably lower for the carbonated (FePC) samples at both ages. The hydration degree of ferrite in FePC_0h was 12%–20% higher than in carbonated samples at 28 days, which may indicate that the reactivity of ferrite did not improve during carbonation but rather slowed down. However, with carbonated samples, FePC_72h had the highest DOH of ferrite at 28 days. Siliceous hydrogarnet phase was not detected in the XRD probably due to its minor quantity and maybe poor crystalline structure [42].

#### TGA

**Fig. 8:**
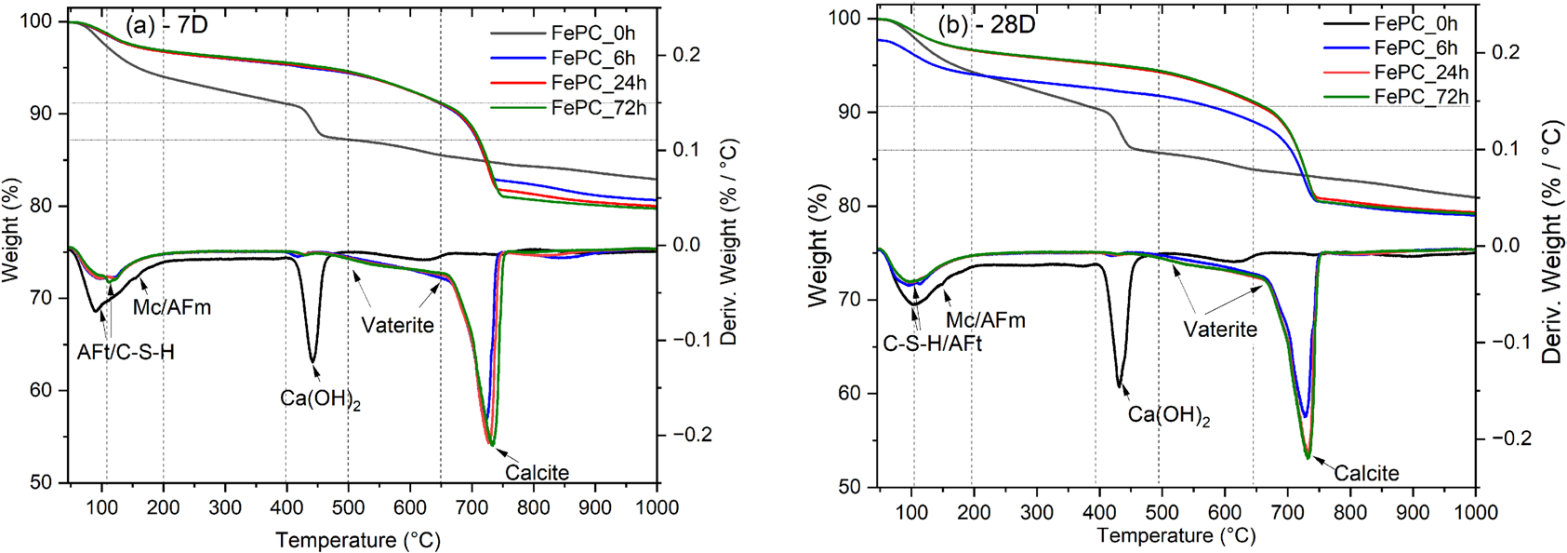
TG/DTG of non-carbonated and carbonated FePC paste samples at (**a**) 7-days and (**b**) 28-days. (For reading of the references to color in figures, the reader is referred to the web version of this article)

The thermal decomposition curves of the hydrates and carbonates in the paste samples at 7-days and 28-days are shown in Fig. 8a and Fig. 8b respectively. The mass loss of amorphous calcium silicate hydrate (C-S-H) phases can be distinguished with the peak between 40–200 °C assigned to the loss of non-evaporable water bound in the C-S-H gel interlayer and its dehydroxylation [43]. This peak overlaps with the dehydroxylation of the ettringite phase [44]. The depth of these peaks varies with the effect of carbonation, as the C-S-H dehydroxylation peaks for FePC_0h sample at both days increased. While the dehydroxylation peak intensity for the carbonated samples lowered, the remaining peak can be assigned largely to formed C-S-H after carbonation from unhydrated cement grains during curing.

CH formation is characterized by the peak and mass losses between the temperature range 400–500 °C for FePC_0h paste sample, while carbonization of CH was confirmed by the diminished peaks in the carbonated sample curves. The carbonation of C-S-H forms vaterite and then calcite [45]. Vaterite an unstable polymorph of calcium carbonate has a lower thermal decomposition due to its lower thermodynamic stability compared to calcite, has thermal decomposition peak between 500 and 700 °C [46]. Decomposition in this range was detected mainly in carbonated samples, and slightly in the FePC_0h sample curves. In addition, carbonated samples show a mass loss with a peak around 700–800 °C, assigned to the decarbonation of calcite formed in the paste.

#### SEM

**Fig. 9:**
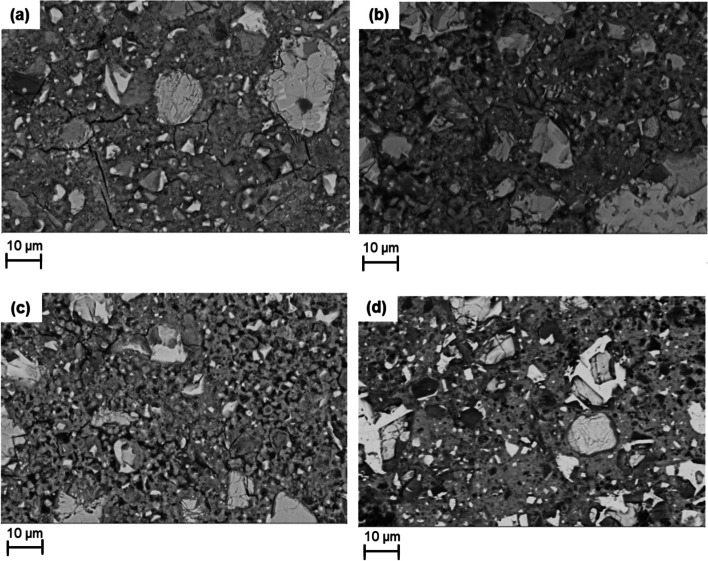
SEM BSE images of the hydrated paste at 28 days (**a**) FePC_0h (**b**) FePC_6h (**c**) FePC_24h, and (**d**) FePC_72h

**Fig. 10:**
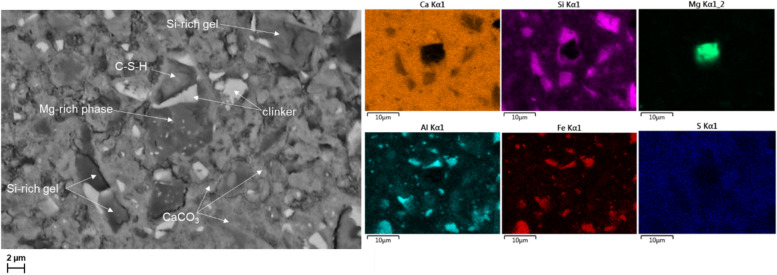
BSE image and elemental distribution maps of Ca, Si, Mg, Al, Fe and S, in the hydrated FePc_72h at 28 days

Figure 9 shows the microstructure of the hydrated samples at 28 days. Using grey-level segmentation for the SEM-BSE images, a distinct contrast in the phases in the microstructure can be observed. Bright areas corresponds to unreacted cement clinker grains, light grey region corresponds to calcium hydroxide, calcium carbonate and C-S-H, and dark areas depicts pores or silica gels [37]. The images show coarse structures and shrinkages after carbonation especially for FePC_6h and FePC_24h when compared with the FePC_0h sample image. Whereas FePC_72h shows interconnected structure. Cracks seen in FePC_0h micrograph may have been due to the methods used during sample polishing. With FePC_72h, darker rims can be seen in the micrographs around the cement grains, consistent with decalcified hydrates [47]. In addition, darker regions of hydrates can be seen in the microstructure. SEM elemental distribution map was acquired to determine these distinct dark areas for FePC_72h to get information about these products’ composition (Fig. 10).

The EDX elemental maps show both homogenous and heterogenous distribution of the elements in the paste (Fig. 10). The SEM–EDX map for Si exhibited darker distribution with what appears to be specifically decalcified silica-rich phase from the decalcification of C-S-H. A single Mg-rich area possibly Mg(OH)_2_ or MgO can be observed in the sample. The elemental distribution also showed Ca-rich areas were predominant in the analysed region. The same distribution was observed with the elemental map of S and may suggest intermixed of hydration or carbonated phases. On the fate of dissolved ferrite, the formation of Fe-containing phases was not distinguishable in the area analysed.

### Leaching and Cr(VI) determination

Using the anhydrous FePC and standard limits as reference, the effects of hydration and carbonation of FePC on leachates are shown in Table 7. Overall, all the measured components were below standard leaching limits for non-hazardous materials [48]. However, results show that increasing carbonation exposure time of FePC increased the leaching of Cr, Mo, V, and SO_4_. The increased leaching of the transition metals ions in the carbonated FePC likely stems from a pH decrease following carbonation, which has the tendency to dissolve chemically bonded metals ions in hydration products like C-S-H, rendering these ions soluble and mobile to be leached [49]. Similarly, sulfate leaching with increasing carbonation exposure may be probably due to carbonation of ettringite as earlier discussed in the XRD section. Via this carbonation reaction, gypsum, which is more soluble than ettringite is produced [50–52].

**Table 7:** Leachable amount of measured elements in clinker, after hydration, and carbonation at 0 h,6 h,24 h and 72 h FePC. The leaching limit values and acceptance criteria for non-hazardous waste in landfills are used as control limits [48]

Measured components	FePC (ppm)	FePC_0h (ppm)	FePC_6h (ppm)	FePC_24h (ppm)	FePC_72h (ppm)	Limit value (ppm)
As	< 0.01	< 0.01	< 0.01	< 0.01	< 0.01	2
Ba	16	4	1.7	0.99	0.72	100
Cd	< 0.002	< 0.002	< 0.002	< 0.002	< 0.002	1
Cr	0.12	0.26	0.63	1.4	2.1	10
Cu	< 0.01	< 0.01	< 0.01	< 0.01	0.012	50
Mo	0.016	< 0,01	0.018	0.031	0.036	10
Ni	< 0.01	< 0.01	< 0.01	< 0.01	< 0.01	10
Pb	0.015	0.0064	< 0.004	< 0.004	< 0.004	10
Sb	< 0.01	< 0.01	< 0.01	< 0.01	< 0.01	0.7
Se	< 0.04	< 0.04	< 0.04	< 0.04	< 0.04	0.5
V	< 0.01	< 0.01	< 0.01	< 0.01	0.017	-
Zn	0.048	< 0.04	< 0.04	< 0.04	< 0.04	15
Hg	< 0.004	< 0.004	< 0.004	< 0.004	< 0.004	0.2
F	< 5	< 5	< 5	< 5	< 5	150
Cl	26	27	28	28	28	15 000
SO_4_	< 50	< 50	< 50	140	470	20 000
pH	12.81	12.44	12.32	12.11	12.01	-
Cr(VI)^a^	0.25	-	-	-	-	-
TDS	24,000	20,000	13,000	8900	7600	60 000

*TDS* Total dissolved solids

^a^Cr(VI) in FePC was determined based on procedures in SFS EN 196–10

Meanwhile, Ba and Pb were immobilized with an increasing degree of carbonation. Similar results on Pb immobilization have been reported in carbonated synthetic Portland cement doped with heavy metals [53]. All measured components were below the permissible limit value.

### MIP

**Fig. 11:**
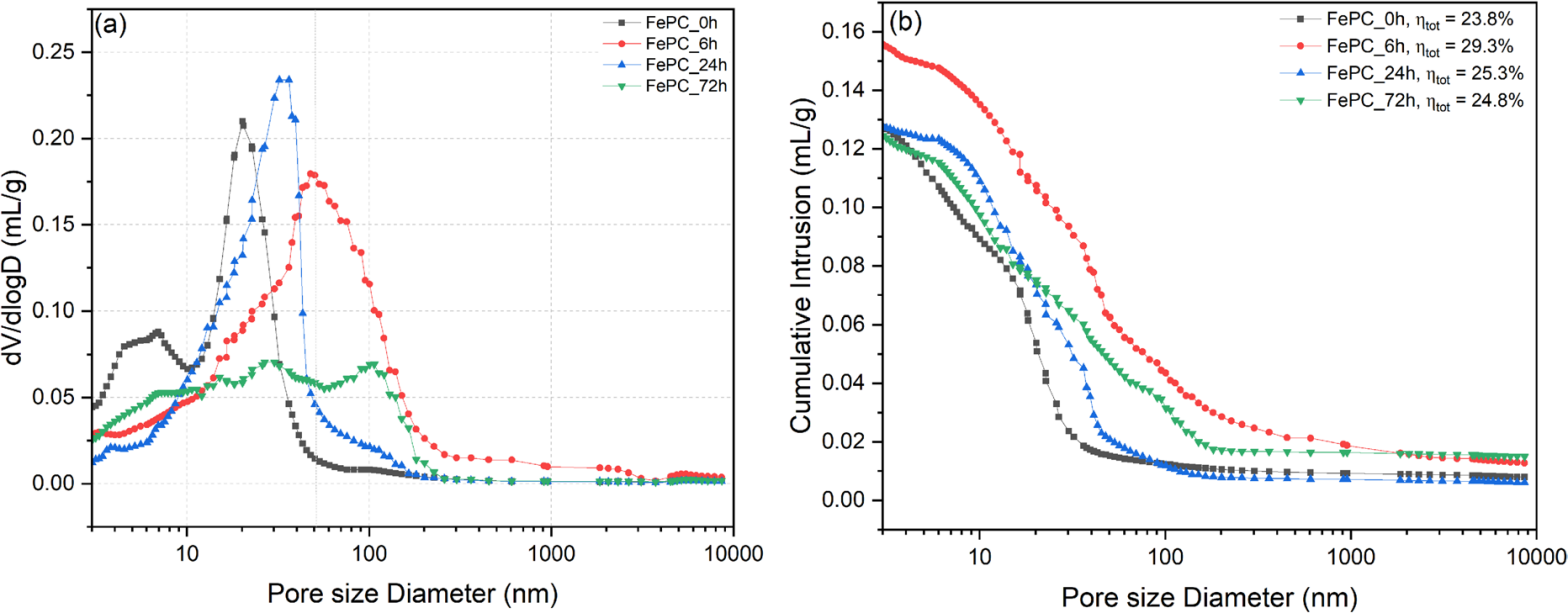
Mercury intrusion curves for the non-carbonated and carbonated paste sample at 28 days (**a**) differential pore size distribution and (**b**) cumulative intrusion curve. Where dV/dlogD is differentialpore volume and n_tot_ is the total intrudable porosity

A clear shift in the pores structure range of the samples can be recognized after carbonation (Fig. 11). The precipitation of calcium carbonate after CO_2_ mineralization in the pore structure refined the samples with higher pore sizes observed with increasing carbonation exposure time and reduction in the pore volume of both big and small capillary pores. The FePC_0h samples are typically characterized by pores with diameter (d) within the range of 10 < d < 50 nm, whereas silica gel has large pores, within the range 100 < d < 1000 nm [54]. This is in line with the observed pore refinement in carbonated samples, indicating the presence of silica gel. Indeed in cement chemistry, it is understood that the carbonation of C-S-H involves its decalcification where calcium is removed to form calcium carbonates and formation of the amorphous silica gel [55]. However, the porosity of the carbonated samples does not linearly increase with time of carbonation curing (Fig. 11) and the FePC_0h sample had lower total intrudable porosity than all the carbonated samples. This inconsistency needs to be interpreted with caution when used in discussing other results. Carbonation of CH to form calcium carbonate is thought to alter the cement pore structure by filling the pores due to the higher solid volume of the carbonate than CH [55]. Thereby the total intrudable porosity is reduced (Table 8) with increasing carbonation exposure time enhanced by the increasing densification of the paste during prolonged carbonation.

**Table 8:** The properties of the FePC mortars exposed to different carbonation times (total intrudable porosity was measured via MIP for 28 days paste samples)

	Compressive strength		
	7-day CS (MPa)	28-day CS (MPa)	Total porosity (%)
FePC_0h	39.0 ± 0.9	50.2 ± 2.9	23.8
FePC_6h	44.0 ± 2.8	59.3 ± 1.8	29.3
FePC_24h	46.1 ± 2.6	51.9 ± 1.7	25.3
FePC_72h	52.7 ± 1.3	59.0 ± 2.1	24.8

### Compressive strength

The compressive strengths and total intrudable porosity of the FePC mortars with and without accelerated carbonation are shown in Table 8. The FePC_0h mortar exhibited an early strength of 39 MPa and a 28-day strength of 50 MPa which conforms with the mechanical requirements and strength class of middle strength category e.g. CEM I 42.5N. After carbonation and further moist curing, the FePC mortars at all testing ages demonstrated higher strength gain, which affirms the further hydration of unhydrated and uncarbonated cement in the matrix after exposure to carbonation. Compared to the FePC_0h mortar, the FePC_72h mortar exhibited a significant 36% increment in compressive strength at 7-days, an effect of accelerated carbonation on the microstructure of the matrix. While at 28-days, the strength gain of FePC_6h and FePC_72h mortars are comparable. The strength of FePC_6h may be attributed to increased hydration of unreacted cement within the mortars during water-curing. It is conceivable that the amount of unreacted cement minerals i.e. C_3_S to be more in FePC_6h after carbonation than at 72 h. Noticeably, a lower strength was achieved for FePC_24h at 28-days compared with other carbonated FePC mortars.

## Further discussion

The clinker produced at 1400 °C from the raw mix of EAF slag (20%), calcium carbonate (67%), kaolin clay (5%) and silicon oxide (7%) lead to FePC clinker with composition of alite (47%), ferrite (31.8%), belite (19.7%), and minor elements CaO and MgO. According to SEM–EDX analysis the clinker also contained minor fractions of synthetic compound Ca-Al–Mg-O phase and monocalcium aluminate (CA). The majority of ferrite phase observed with SEM–EDX has chemical composition close to stoichiometric brownmillerite (C_4_AF) but minor amounts of low iron containing ferrite was also found.

**Fig. 12:**
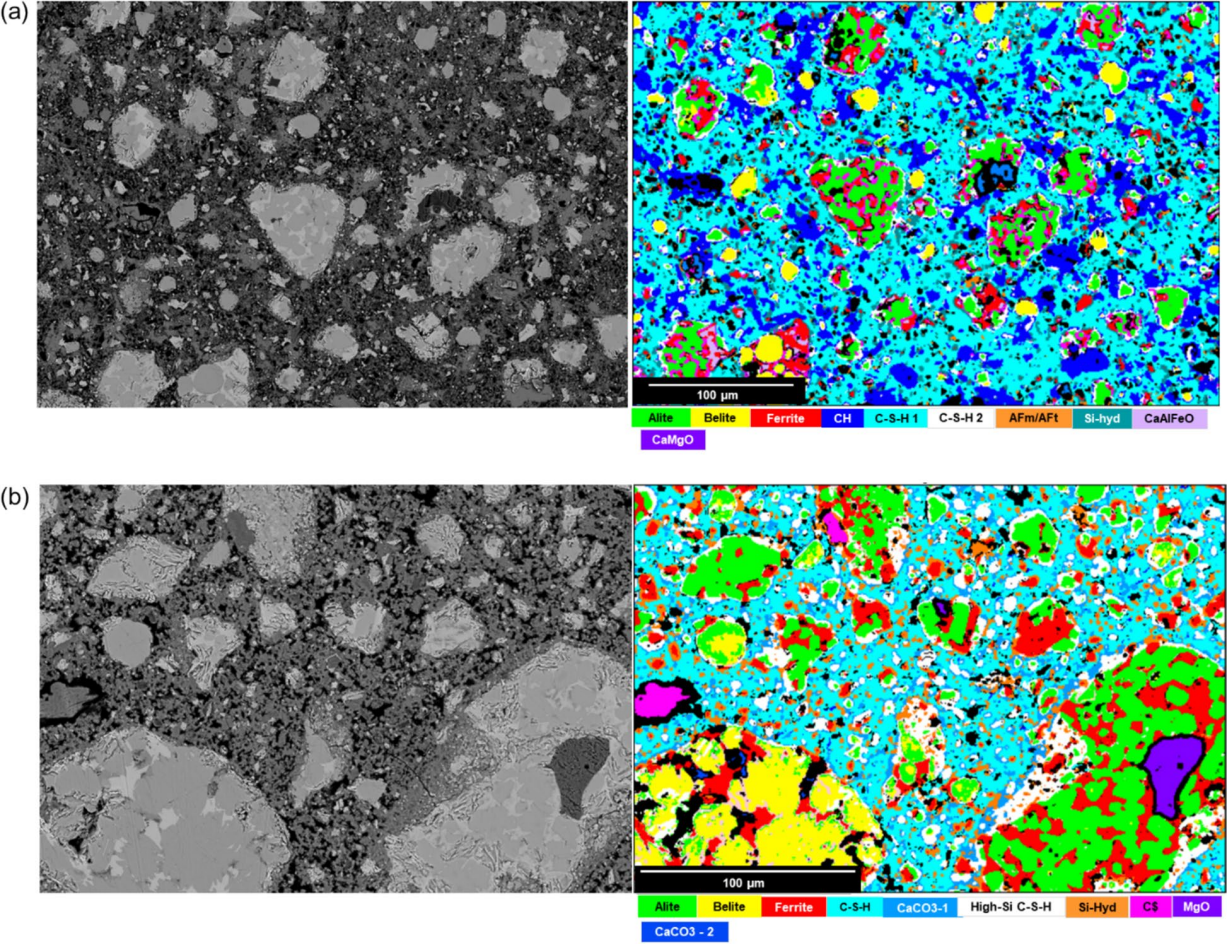
BSE image (left) and phase elemental maps (right) of polished sections of (**a**) FePC_0h and (**b**) FePC_72h pastes

The early hydration of produced clinker was found to be slow. The addition of gypsum in the cement enhanced the early hydration and formation of hydration products as seen in the isothermal calorimetry test. To further validate the results from Sect. 3.3, the EDX phase maps of the hydrated and carbonated samples were acquired (Fig. 12). The Backscattered electron (BSE) images for FePC_0h and FePC_72h and the phase mappings show a complex distribution of unhydrated, hydrated, and carbonated phases. The microstructure shows distinct areas of unhydrated phases of alite, ferrite and belite. Figure 12a displays the four differentiated hydration products (C-S-H phases, AFm/AFt, CH and siliceous hydrogarnet like-phases) for FePC_0h. Distinctly, C-S-H 1 is the main hydration product deposited in between the unhydrated particles and comprises intergrown nanoscale hydration products such as C-S-H, Aft/AFm and also Calcium Hydroxide (CH). The oxide composition of this phase is shown in Fig.S3. The incorporation of Fe^3+^ ions has been reported in the interlayer of C-S-H phases with high Ca/Si [56]. Differentiated from C-S-H 1 in the EDX phase map is C-S-H 2, forming a rim around alite particles. The formation of siliceous hydrogarnet (Si-hyd) from ferrite hydration can be seen finely distributed within the hydrate matrix and in vicinity of ferrite. This phase has been reported to form in Fe-rich cements [27, 36, 57], and contains sizable amount of Fe in its elemental composition (See Fig.S4). This identification suggests a certain degree of hydration of the ferrite phases. Noticeably, this phase also overlaps or forms cluster with the AFt/AFm phase in the map. In a study by Dilnesa et al. [27], Al/Fe-siliceous hydrogarnet was found to be more stable than Fe-containing AFm phases and Fe-ettringite in OPC. Lothenbach et al. reported that only Fe-siliceous hydrogarnets is the most stable Fe-phases in Fe-containing hydrated cement [57]. They further stated that during the first hours of hydration, Fe (III) precipitates as FeOH and later as siliceous hydrogarnets.

In cement chemistry, the hydration of alite to form CH is suggested to slow down the reactivity of the ferrite phase due to high saturation of Ca^2+^ in the matrix and formation of protective layer around ferrite particles in the early days of hydration [58]. By carbonating the CH and potentially the protective layer to calcite and under saturating the Ca^2+^ in the matrix, the reactivity of ferrite phase is theorized to increase while calcite acts as seeding sites [38]. The EDX phase map of hydrated FePC_72h (Fig. 12b) likewise reveals the presence of hydrates such as C-S–H, siliceous hydrogarnet, AFm/AFt, and gypsum. One of the C-S-H (high Si/Ca) phases observed is characterized by high silica content which might be a result of C-S-H carbonation [37] (see Fig.S3). This is supported by the calcium carbonate rim around this phase in Fig. 12b. Again in the vicinity of the unhydrated ferrite siliceous hydrogarnet is revealed. The identification of pure siliceous hydrogarnets remains difficult, most likely the hydrogarnet is of sub-micron scale, and therefore the EDX mapping resolution is unsuited to detect pure hydrogarnet. The oxide composition of this Si-hyd phase (Fig. 12b) shows composition of Ca, Si, Fe, Al, S (Fig.S5). It is likely and consistent with findings from previous study [59], that the area analyzed contains sub-micron scale AFm/AFt. This phase intergrowth at the lower micron scale leads to deviations in oxide compositions from pure phase but is unavoidable. Consistent with the observation seen in XRD (Fig. 6) and reported in previous studies [40, 41, 60], gypsum is observed after carbonation of ettringite.

**Fig. 13:**
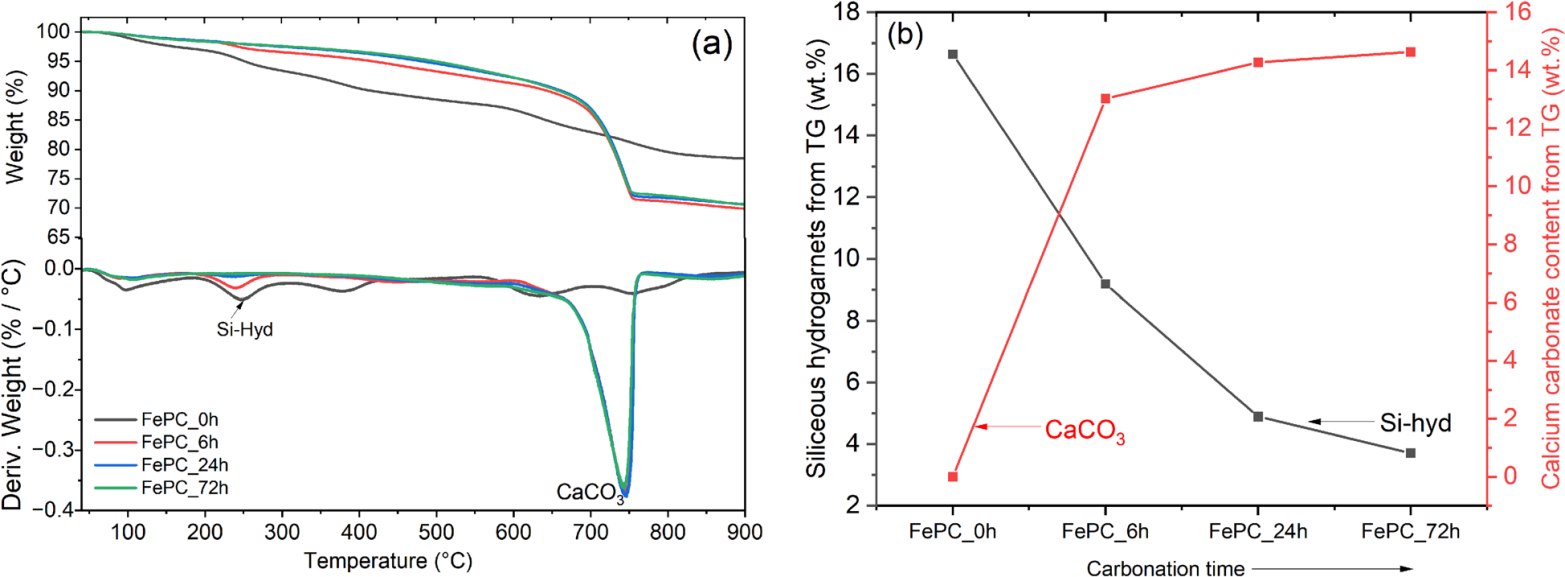
**a** TG-DTG curves of the paste samples after SD, and (**b**) Determined amount of Si-hyd after SD, the CaCO_3_ was calculated before SD

Selective dissolution (SD) technique was performed to extract the main components of the hydrated paste samples, while the minor phases formed during the hydration process such as siliceous hydrogarnet (Si-hyd) can be identified. The residual paste was characterized using TG/DTG to identify and quantify the minor phases. In Fig. 13 shows the TG-DTG curves of the paste after selective dissolution (SD), results reveal that increasing carbonation time decreased the Si-hyd content. The reason might be due to too much formation of CH from alite, which converts to CaCO_3_ on carbonating. The early carbonation of CH, possibly delayed the ferrite phase hydration due to the densification of the microstructure impeding the ingress of CO_2_ to the surface area of the ferrite phase (see Fig. 12). And can be attributed to the reduced dissolution of the ferrite phase with increasing carbonation, thus seems to be the case as the determined content of Si-hyd is consistent with the degree of ferrite hydration (Fig. 7c). Perhaps having lower amount of alite in the cement clinker and more belite can potentially influence this expected mechanism.

## Conclusions and outlook

The synthesis of ferrite-rich cement clinker using EAF slag as alternative raw material was investigated as one of the pathways for utilization of underused industrial byproduct and to reduce the carbon footprints of the cement production industry. To improve the reactivity of the ferrite phase, we investigated the effect of carbonation at different exposure times on the phase assemblage. Based on the experimental results discussed, the following conclusions can be stated as summarized below:

- The clinker synthesis shows that it’s possible to replace 20% of clinker starting materials with EAF slag to produce alite (47%) and ferrite (32%)-rich cement. Utilizing EAF slag in the clinker production has the potential to reduce the production temperature (< 50 ℃) and therefore the energy consumption, replace or substitute all iron ore required in making FePC and partially replace limestone (18%), saving carbon emissions and utilization of natural resources.
- By adding 5% of gypsum to the cement clinker, the hydration rate of the phases (i.e. alite, ferrite) and formation of portlandite and ettringite as determined via isothermal calorimetry were enhanced compared to cement clinker without gypsum. This hydration acceleration was ascribed to the increased formation of ettringite (possibly Fe-ettringite) and enhancement of alite hydration.
- Portlandite, C-S-H, Fe/Al-rich ettringite and siliceous hydrogarnet were the main phases formed in the hydrated FePC.
- The hydration of the ferrite phase formed Fe/Al-rich siliceous hydrogarnets and ettringite. The formation of the former was not enhanced on exposure to accelerated carbonation, which may be due to the early carbonation of CH, delaying the ferrite phase hydration due to the densification of the microstructure impeding the ingress of CO_2_ to the surface area of the ferrite phase. While ettringite decomposes on prolonged carbonation exposure to gypsum.
- With the decomposition of the ettringite phase after carbonation, the leaching of Cr, Mo, V, and SO_4_ increased.

The ferrite phase is an important phase within cement clinkers, with documented effects on sulfate resistance and hydration characteristics, consequently enhancing the durability of hydrated cement. Understanding the hydration of the ferrite phase is key if iron-rich byproducts are to be utilized as raw materials for cement clinkers. The presence of Cr, Mo, and other minor elements in steelmaking slags presents challenges for clinker producers, standardization committees, and lawmakers, urging them to adapt if conventional raw materials are to be replaced with steelmaking slags. The strength of produced cement falls just under the category of high strength concrete. Due to high density of ferrite rich cement renders it suitable for application in radiation shielding, structure foundations, oil-well cementing, and roadblocks. Further research is desired in further lowering the clinker production conditions and enhancement of the ferrite phase reactivity in the systems with belite-ferrite and alite-belite-ferrite.

## Supplementary Information

Supplementary Material 1.

## Data Availability

Data will be made available on request.
