# Supplementary material for: Hydration and carbonation curing of high ferrite clinker (FePC) synthesized using EAF slag
Source: Low Carbon Mater Green Constr. 2024 Dec 4;2(1):21. doi: 10.1007/s44242-024-00051-9 (PMC11698376; doi:10.1007/s44242-024-00051-9)
Supplement: Supplementary file 1 — Supplementary Material 1. [file 44242_2024_51_MOESM1_ESM.docx]

## **Supplementary**


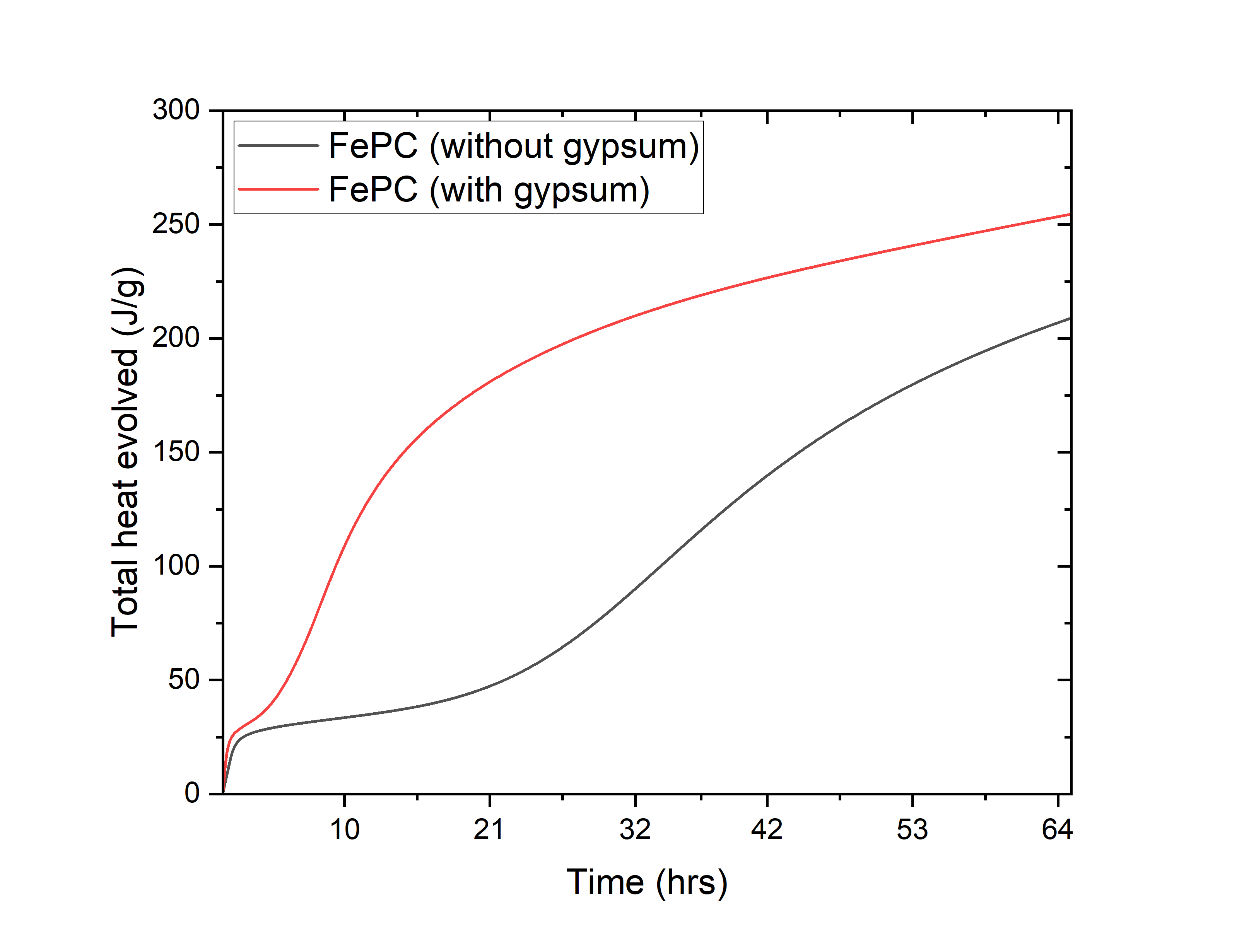


#### Fig. S1. Cumulative heat released in FePC paste with and without gypsum.


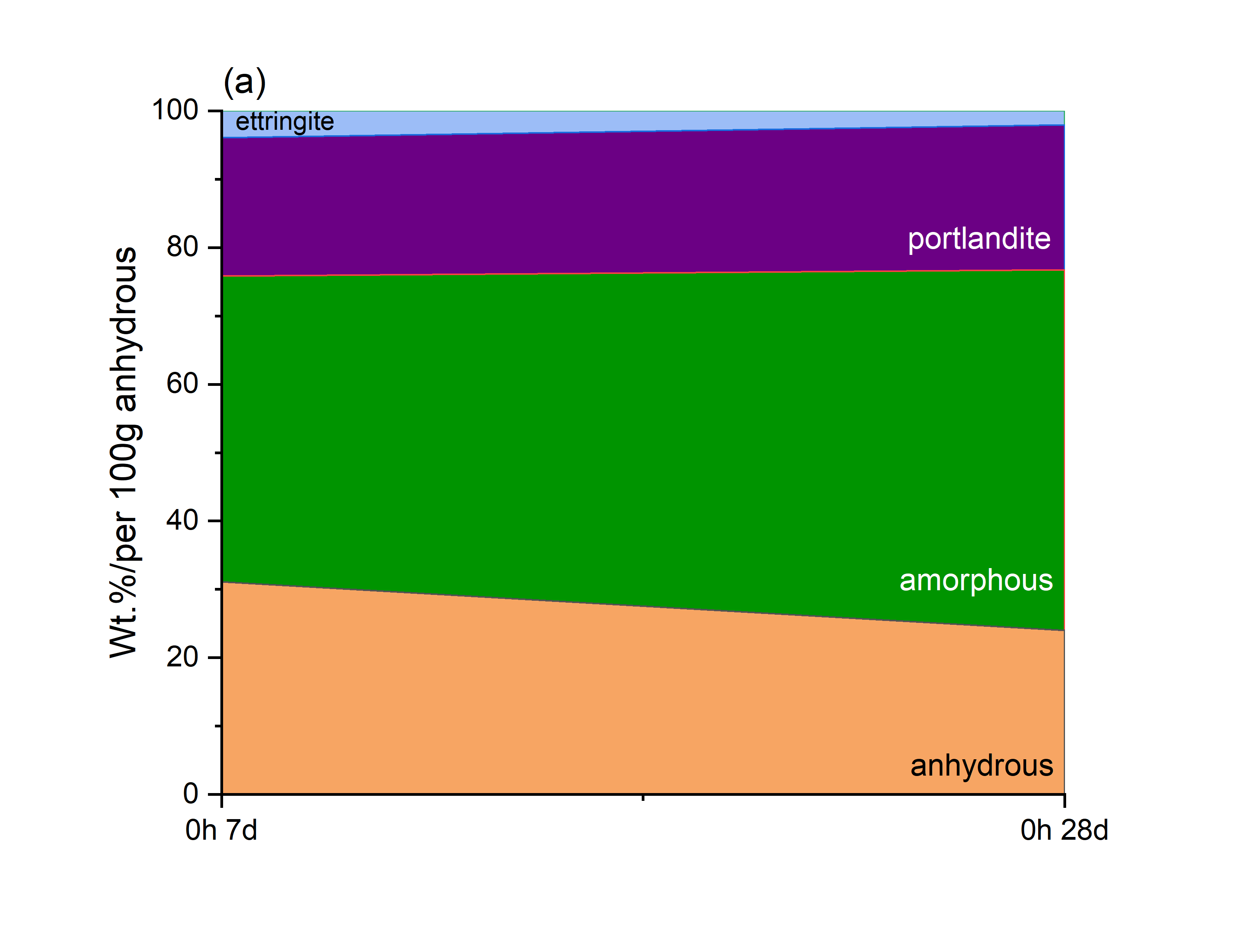

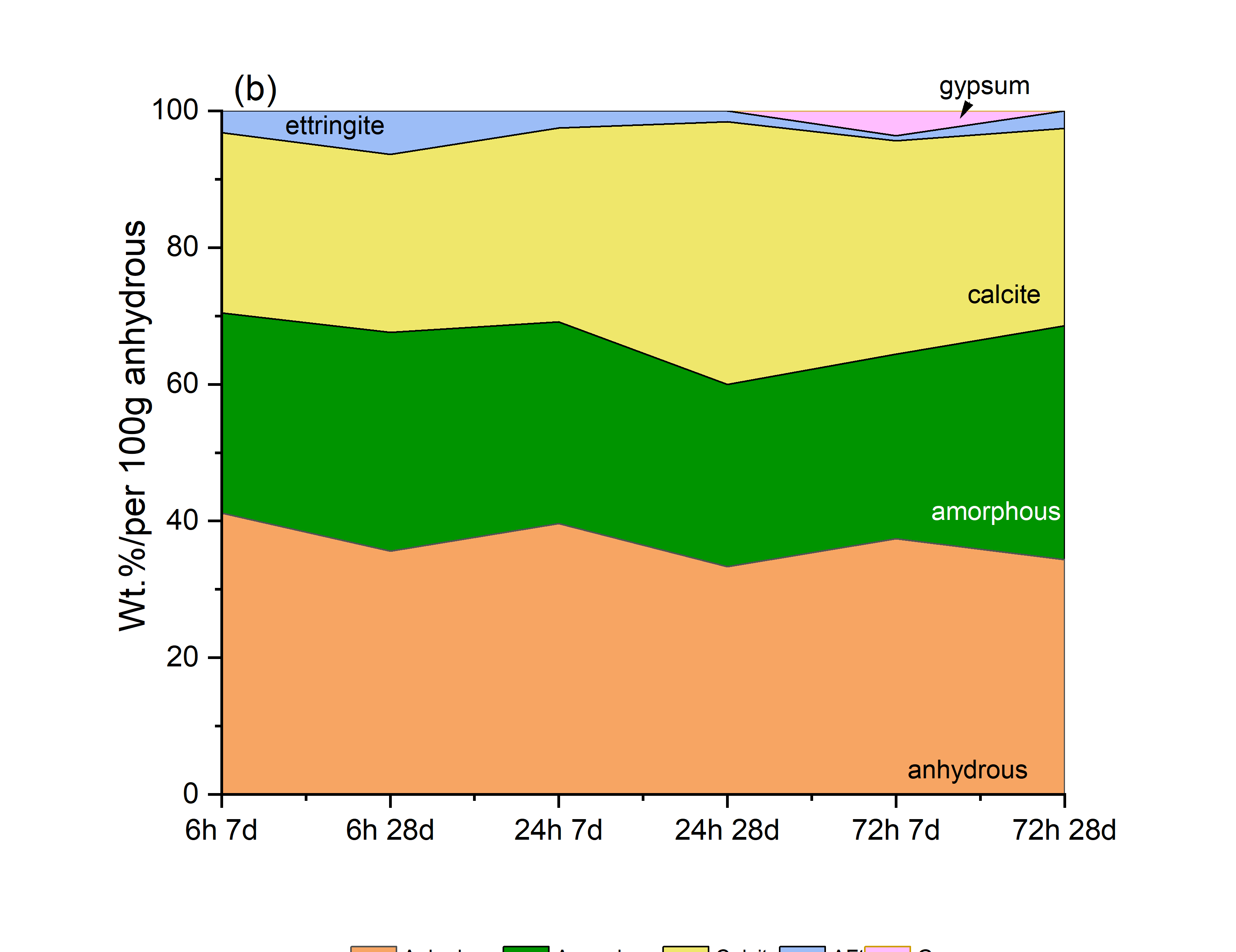


#### Fig. S2. Quantified weight content of unreacted/anhydrous and formed hydration and carbonation products content at 7 and 28 days (a) non-carbonated FePC pastes and (b) carbonated FePC pastes.


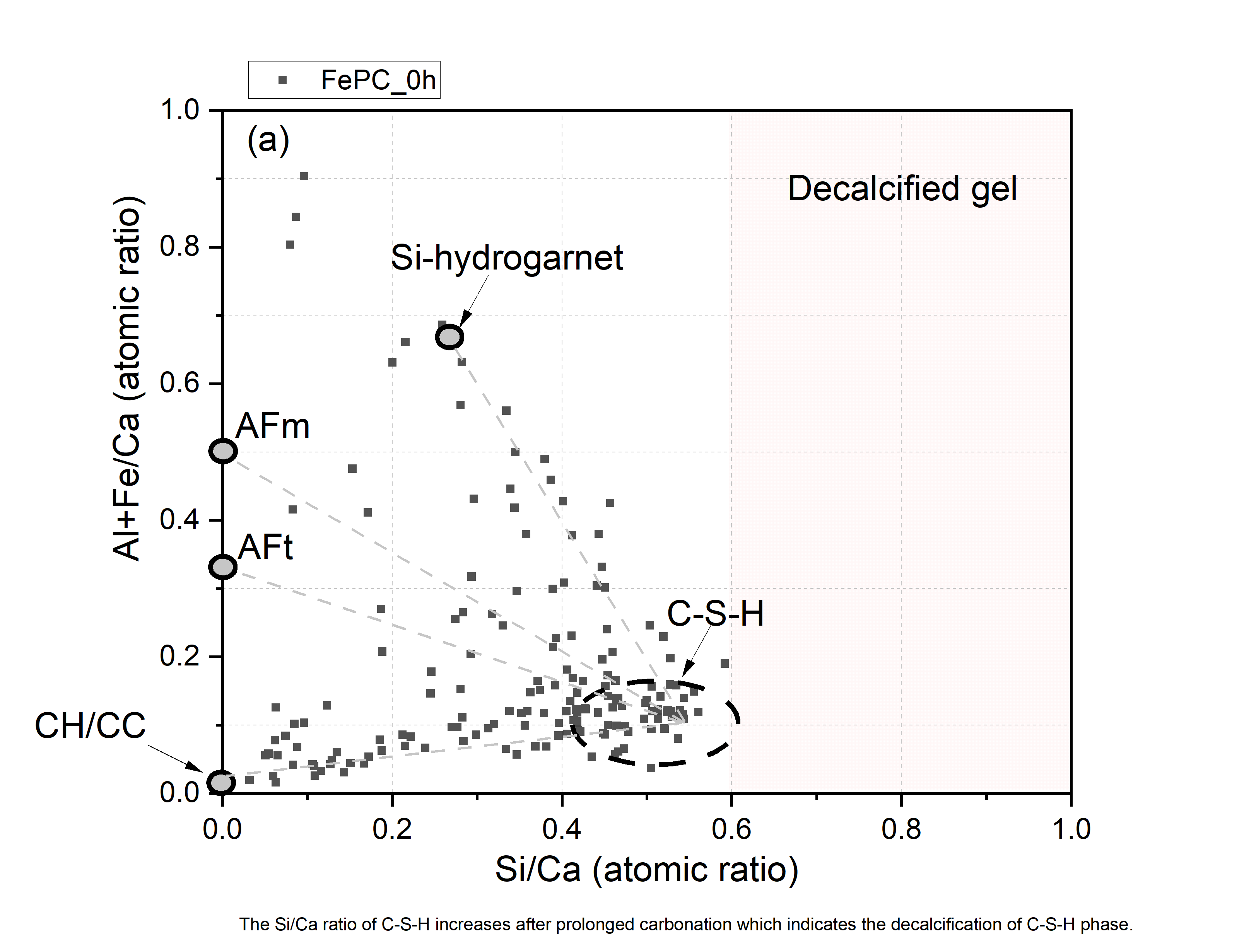

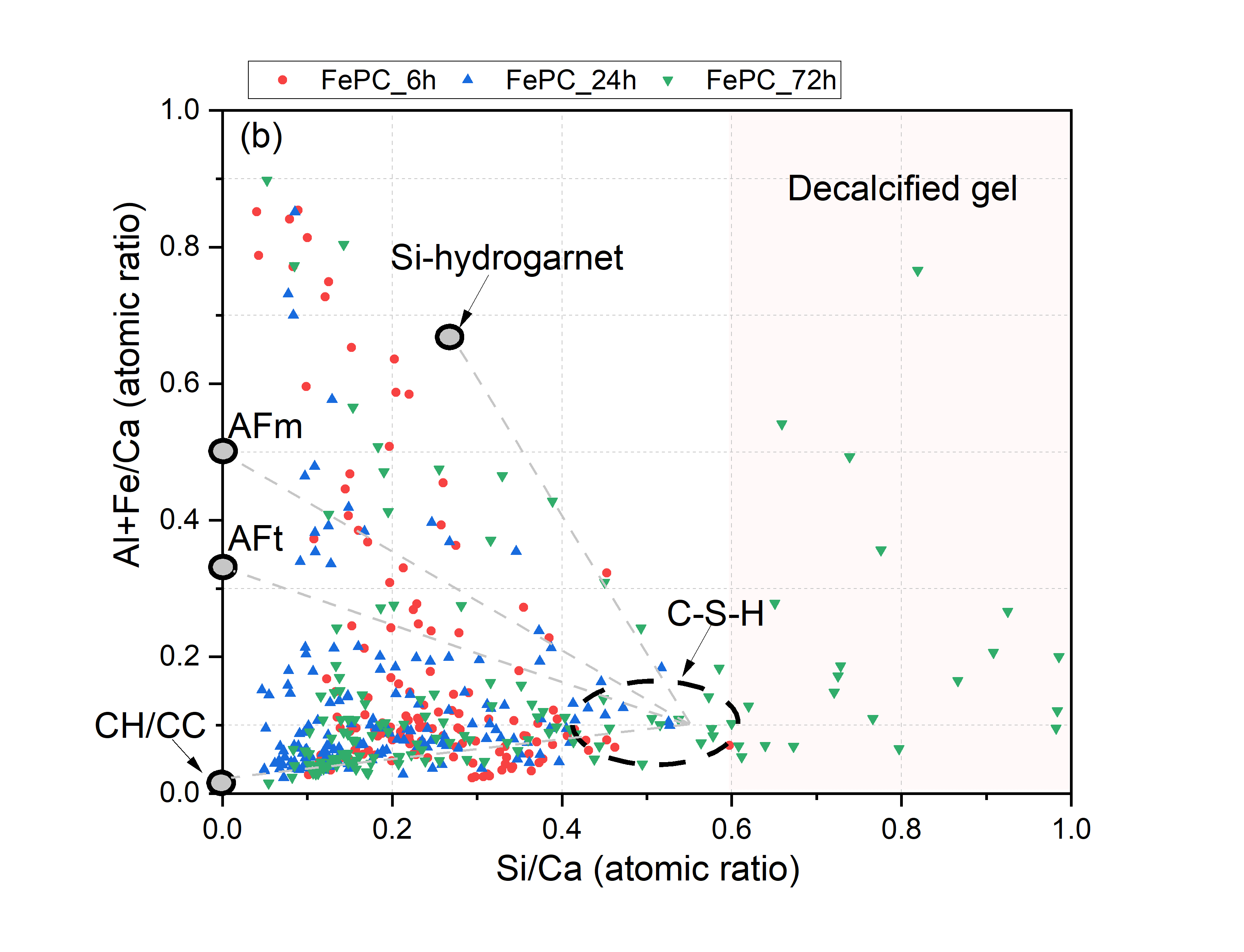


#### Fig. S3. 2D scatter plots of the atomic ratio (Si/Ca versus Al+Fe/Ca) of selected hydration product points at 28 days. AFm: monosulfate, AFt: ettringite, CH:portlandite, CC:calcium carbonates, and C-S-H.


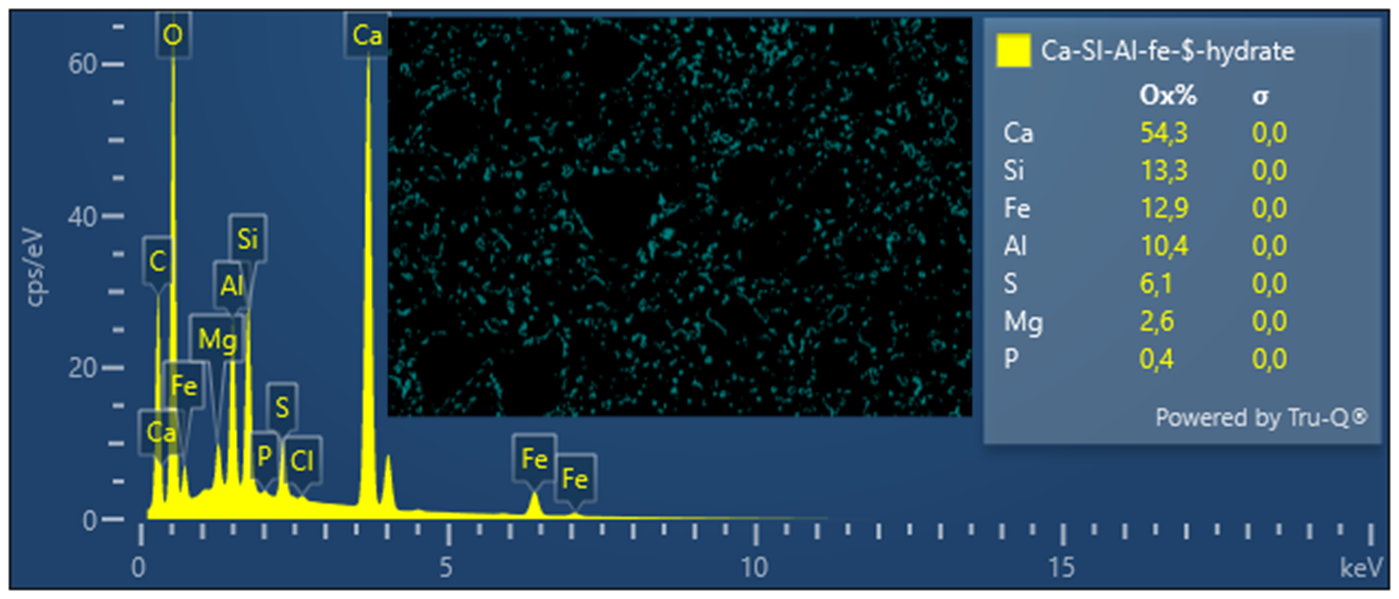


#### Fig. S4. Elemental spectra of segmented Fe/Al-siliceous hydrogarnet phase map from [Fig.11a](#_Fig.11._Phase_maps) (FePC_0h).


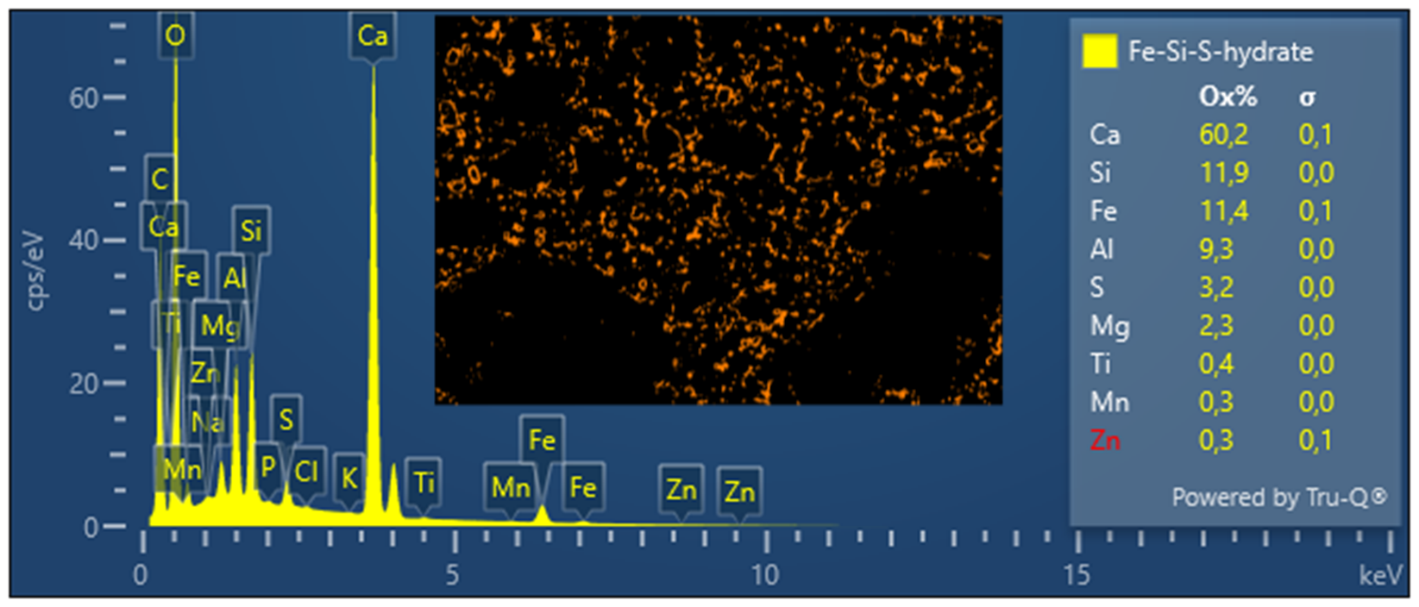


Fig. S5. Elemental spectra of segmented Fe/Al-siliceous hydrogarnet phase map from [Fig.11b](#_Fig.11._Phase_maps) (FePC_72h).
